# Neuronal On- and Off-type heterogeneities improve population coding of envelope signals in the presence of stimulus-induced noise

**DOI:** 10.1038/s41598-020-67258-1

**Published:** 2020-06-23

**Authors:** Volker Hofmann, Maurice J. Chacron

**Affiliations:** Department of Physiology, McGill University, Montreal, QC Canada

**Keywords:** Neural encoding, Computational neuroscience, Sensory processing

## Abstract

Understanding the mechanisms by which neuronal population activity gives rise to perception and behavior remains a central question in systems neuroscience. Such understanding is complicated by the fact that natural stimuli often have complex structure. Here we investigated how heterogeneities within a sensory neuron population influence the coding of a noisy stimulus waveform (i.e., the noise) and its behaviorally relevant envelope signal (i.e., the signal). We found that On- and Off-type neurons displayed more heterogeneities in their responses to the noise than in their responses to the signal. These differences in heterogeneities had important consequences when quantifying response similarity between pairs of neurons. Indeed, the larger response heterogeneity displayed by On- and Off-type neurons made their pairwise responses to the noise on average more independent than when instead considering pairs of On-type or Off-type neurons. Such relative independence allowed for better averaging out of the noise response when pooling neural activities in a mixed-type (i.e., On- and Off-type) than for same-type (i.e., only On-type or only Off-type), thereby leading to greater information transmission about the signal. Our results thus reveal a function for the combined activities of On- and Off-type neurons towards improving information transmission of envelope stimuli at the population level. Our results will likely generalize because natural stimuli across modalities are characterized by a stimulus waveform whose envelope varies independently as well as because On- and Off-type neurons are observed across systems and species.

## Introduction

**Figure 1:**
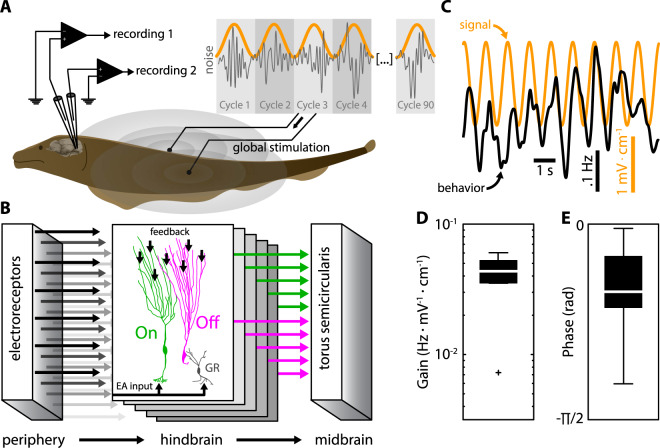
Experimental setup and behavioral responses to electrosensory stimulation. **(A**) Recordings were made during stimulation with spatially uniform (‘global’) stimuli mimicking those encountered during interactions with a conspecific. Inset: Stimuli consisted of modulations of the self-generated EOD (not shown). Specifically, stimuli were constructed as non-repetitive low-pass filtered (15 Hz cutoff) Gaussian white noise (i.e., the “noise”; gray) whose amplitude, varied sinusoidally with frequency 1 Hz, (i.e., the “signal”; orange). The gray shading shows each cycle of the signal. (**B**) Electrosensory stimuli are sensed by electroreceptor afferents (EAs, left) on the skin that project to pyramidal neurons in the hindbrain electrosensory lateral line lobe (ELL, center) that are either of the On- or Off-type variety: On-type neurons (green) receive direct excitatory EA input while Off-type neurons (magenta) receive EA input indirectly via inhibitory granular interneurons (GR). Both project to higher order brain areas such as the midbrain torus semicircularis. (**C**) The signal (orange) elicited behavioral responses (black) that consisted of the animal tracking the stimulus waveform through changes in EOD frequency. (**D,E**) Quantification of behavioral responses to envelopes using linear systems identification techniques from N = 7 fish used in the study. Shown are the gain (D; 0.040 ± 0.017 Hz · mV^−1^ · cm^−1^) and phase (E; −31 ± 23°).

Understanding the set of transformations by which sensory input leads to behavioral responses remains a central problem in neuroscience. It has been widely observed that neurons display heterogeneities, even within the same class^1–6^. Although much effort has focused on understanding how heterogeneities affect population coding^7–12^, considerably less effort has focused on uncovering their role in determining neural responses to the often-complex features of behaviorally relevant stimuli. Here we used the electrosensory system of the weakly electric fish *Apteronotus leptorhynchus* (Fig. 1A) to demonstrate how neural heterogeneities can improve signal transmission at the population level of a behaviorally relevant signal that is embedded in stimulus-induced noise.

Weakly electric fish rely on perturbations of an electric field self-generated around their body through the quasi-sinusoidal electric organ discharge (EOD) in order to sense their surroundings and communicate with conspecifics. Interactions between the EODs of two or more moving conspecifics creates complex electrosensory stimuli consisting of a relatively fast time-varying amplitude modulation of the EOD whose amplitude (i.e., the envelope) varies more slowly^13,14^. The time-varying envelope carries behaviorally relevant information about the distance between conspecifics^13^ and behavioral studies have shown that information about the detailed time course of the envelope is retained within the brain^15^. EOD perturbations are detected by electroreceptors scattered on the animal’s skin surface whose afferents synapse onto pyramidal neurons within the electrosensory lateral line lobe (ELL). There are two main types of ELL pyramidal neurons: On-type cells that respond with increased firing activity to increases in the stimulus as they receive direct excitatory input; and Off-type cells that instead respond with increased firing activity to decreases in the stimulus as they receive indirect inhibitory input from afferents via local interneurons (Fig. 1B)^16–18^. The responses of single ELL pyramidal neurons to the stimulus waveform^19–22^ and the envelope^23–32^ have been extensively characterized. In particular, single neurons will respond to both the stimulus waveform and its envelope^14,23,30^. However, how ELL pyramidal neurons encode envelopes at the population level, which is most likely required to elicit the observed behavioral responses, has not been investigated to date.

Here we investigated how ELL pyramidal neural populations encode envelope signals. To do so, we focused on the fact that envelopes are independent of the underlying stimulus waveform^13,33^. As such, we considered the envelope to be the signal while the stimulus waveform was considered to be noise and will henceforth refer to the envelope as the signal and the stimulus waveform as the noise. Our results show that heterogeneities in the ELL pyramidal neuron population help make their responses to the noise more independent, which allows for better averaging out when pooling neural activities and thus greater information transmission about the signal, as compared to that obtained for more homogeneous neural populations.

## Results

We recorded the activities of n = 41 ELL pyramidal neurons from awake behaving animals. Specifically, our stimuli impinged on most of the sensory epithelium in a uniform fashion (Fig. 1A). ELL recordings were categorized into On- and Off-type using an AM stimulus (i.e., 0–120 Hz noise) that was independent of that used throughout the remainder of our study (see methods). Consistent with previous results, On-type cells typically responded during the AM stimulus upstrokes (Supplementary Fig. S1A, green) while Off-type cells typically responded during the AM stimulus downstrokes (Supplementary Fig. S1A, magenta). The spike triggered averages (i.e., the average AM stimulus waveform preceding the action potential) from On-type cells displayed positive slopes before the action potential (Supplementary Fig. S1B) while those of Off-type cells instead displayed negative slopes (Supplementary Fig. S1C). We thus computed the average slope of the spike triggered average during a time window preceding the action potential (see Methods) and found a clear bimodal distribution (Supplementary Fig. S1D). Based on this classification, our dataset consisted of n = 21 On-type and n = 20 Off-type neurons.

Our study focused on how On- and Off-type ELL pyramidal neurons responded to a noisy stimulus waveform (0–15 Hz, the “noise”) whose amplitude (the envelope or “signal”) varied sinusoidally at 1 Hz (Fig. 1A,B). The sinusoidal signal could be extracted directly from the noise stimulus waveform via standard nonlinear transformations (Supplementary Fig. S2A, brown). By construction, signal and noise were independent of one another (Supplementary Fig. S2B). It is important to note that the signal was behaviorally relevant as it elicited robust behavioral responses during which the animal’s EOD frequency faithfully followed the signal (Fig. 1C–E). Further, behavioral responses were significantly more correlated with the original sinusoidal signal than with the extracted signal (Supplementary Fig. S2C). We note that it is likely that variability in the behavioral responses are due to internal noise within the brain as well as within the electric organ generating the behavior, and that such variability cannot be inferred from the stimulus waveform itself. This result demonstrates that information about the detailed time course of the sinusoidal signal is transmitted by ELL pyramidal neuron populations and retained within higher order brain areas. We thus considered the sinusoidal signal throughout this study and investigated how neural responses to the noise stimulus waveform (i.e., the noise) influence coding of the signal by ELL pyramidal neural populations.

### On- and Off-type pyramidal neuron responses are out of phase with respect to the noise, but in phase with respect to the signal

**Figure 2:**
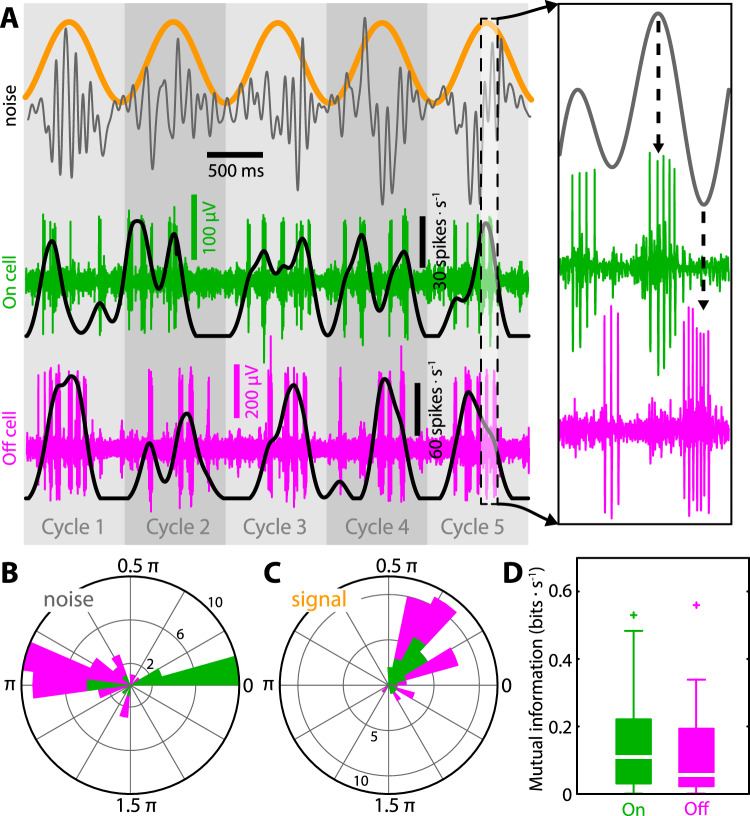
On- and Off-type pyramidal neuron responses to the noise are more heterogeneous than those to the signal. (**A**) Stimulus waveform (top) consisting of a noisy carrier (“noise”; gray) with sinusoidally varying signal (“signal”; orange) and extracellular recorded spiking responses of example On- (middle, green) and Off-type (bottom, magenta) ELL pyramidal neurons. For both neurons, the time-varying firing rates (black traces) obtained by low-pass filtering the spike train with a cutoff of 3 Hz are also shown (see Methods). Spiking responses were out of phase with respect to the noise (see dashed arrows in the inset) but instead in phase with respect to the signal (see main panel, black traces). The gray shading indicates the cycle of the signal. (**B**) Response phase distributions to the noise differed significantly (Kruskal Wallis, p = 2.148 · 10^−5^) between On- (39.0° ± 86.9°; green, n = 21) and Off-type (160.9° ± 45.5°; magenta, n = 20; data is shown as a cumulative histogram such that no datapoints are hidden and each neuron is counted once). We note that the stimulus used to classify cells as either On- or Off-type (0–120 Hz noise; see Supplementary Fig. S1 & methods) was different than that shown in A (0–15 Hz amplitude modulated noise). (**C**) Response phase distributions to the signal were not significantly different from one another (Kruskal-Wallis, p = 0.32) for On- (30.5° ± 42.1°; green) and Off-type (39.9° ± 35.8°; magenta) neurons (data is shown as a cumulative histogram such that no datapoints are hidden and each neuron is counted once). (**D**) Population-averaged mutual information rate (MI) with respect to the signal for On- (0.150 ± 0.150 bits/s; green) and Off-type (0.120 ± 0.145 bits/s; magenta) neurons were not significantly different from one another (Kruskal Wallis, p = 0.51).

Our results show that On- and Off-type pyramidal neurons responded preferentially during either the upstrokes or downstrokes of the noise, respectively (Fig. 2A, inset). As such, the responses of On- and Off-type neurons were approximately out of phase with one another (Fig. 2B). This is expected based on their classification (Supplementary Figs. 1B–D) and previous results^17,18,34,35^. In contrast, On- and Off-type neurons responded with similar increases in firing rate (Fig. 2A, black lines) to increases in the signal. These responses were largely in phase with one another (Fig. 2C), confirming previous results^26,27^. Mutual information rate (MI) values between the single neuron spiking activities and the signal were on average comparable for On- and Off-type pyramidal neurons (Fig. 2D). We note that, while useful to illustrate the phase relationship between responses of ELL pyramidal neurons, low-pass filtering the spiking activities to obtain time-dependent firing rates such as those shown in Fig. 2A will attenuate responses to the noise, which has higher frequency content than the signal by construction. These firing rates are thus shown for illustrative purposes only and are not considered for further analysis.

### Effects of neural heterogeneities on signal and noise response similarity

**Figure 3:**
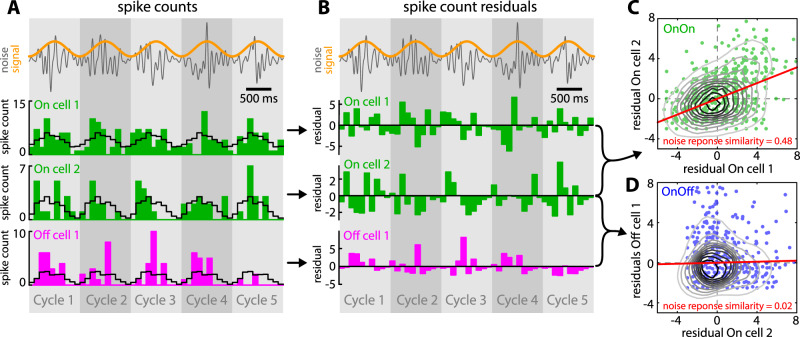
Computing signal and noise response similarity for ELL pyramidal neuron pairs. (**A**) Noise waveform (gray) and signal (orange) with the spike count sequences of two example On-type cells (green) and one Off-type cell (magenta). Cells were recombined from our dataset and not recorded simultaneously. For each cell, average spike count sequence over the envelope cycle (gray shading) is shown (black, plotted as concatenated repetitions). (**B**) Noise waveform (gray) and signal (orange) with the residual spike count sequences (i.e., the spike count minus the average spike count in response to the signal) corresponding to the example neurons shown in A. (**C**) Residual spike counts of On-type cell 2 as a function of that of On-type cell 1 together with levels curves and best-fit straight line (red). The similarity between the residual spike counts (i.e., the noise response similarity) was quantified by the correlation coefficient. For this pair, we obtained a value of 0.48, which was at the higher end of the range observed experimentally. (**D**) Same as C, but for Off-type cell 1 as a function of On-type cell 2. Note the much weaker noise response similarity of 0.02, which was at the median of the range observed experimentally.

We next investigated how heterogeneities in responses to the noise and signal affect coding by pyramidal neuron populations. To do so, spike trains were converted into spike counts using non-overlapping time windows (see Fig. 3A & methods) whose length were smaller than the duration of the envelope cycle (Fig. 3A, gray shading). The similarity between the spike counts sequences from pairs of non-simultaneously recorded spike trains was then quantified using the correlation coefficient (see Methods). Signal response similarity was quantified using the correlation between spike count series that were randomly shuffled according to the signal cycle, thus eliminating the response to the noise which is not repeated across signal cycles (Fig. 3A, gray shading). Noise response similarity was instead quantified using the correlation coefficient between the residual spike counts (Fig. 3B, see methods). Residual spike counts were obtained by subtracting the signal-cycle average (Fig. 3A, black) from each spike count sequence (Fig. 3A, green and magenta), thereby revealing responses to the noise. There are three possible types of pairs: same-type pairs consisting of either two On- (On-On) or two Off-type (Off-Off) neurons, and opposite-type pairs consisting of one On-type and one Off-type neuron (On-Off). Figure 3C,D show the residual spike counts plotted for example same-type (Fig. 3C) and opposite-type (Fig. 3D) pairs. Noise response similarity was positive and large in magnitude for the same-type pair (Fig. 3C) and much closer to zero for the opposite-type pair (Fig. 3D). We note that this is expected based on the fact that the responses of On- and Off-type cells to the noise were maximal at different times during the cycle, which leads to differences in the residuals that attenuate their similarity (Fig. 3A,B).

**Figure 4:**
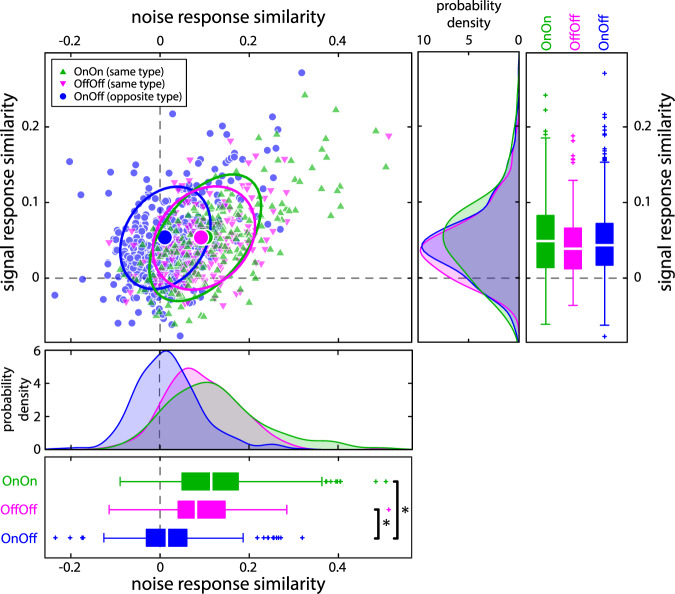
The relationship between signal and noise response similarity depends on cell type. Signal response similarity as a function of noise response similarity for all possible pairs (n = 820 pairs) in our dataset (see Methods). Signal and noise response similarities were computed using a time window length of 100 ms from spike counts and spike count residuals, respectively. Shown are data from same- (On-On: green triangles, n = 210; Off-Off: magenta triangles, n = 190) and opposite-type pairs (On-Off: blue circles, n = 420). The large dots indicate the mean values of signal and noise response similarity for each pair type. The ellipses show the centroid fitted to the 95% confidence level of the two-dimensional distributions. Right: Signal response similarity distributions strongly overlapped for all types of pairs and were thus not significantly different from one another (On-Off, blue: 0.047 ± 0.050; On-On, green: 0.051 ± 0.054; Off-Off, magenta: 0.043 ± 0.043; Kruskal Wallis, p = 0.219). Bottom: Noise response similarity distributions strongly overlapped for On-On and Off-Off pairs (On-On, green: 0.125 ± 0.110; Off-Off, magenta: 0.096 ± 0.082). However, the distribution for On-Off pairs (On-Off, blue: 0.019 ± 0.070) was significantly different from the other two (Kruskal Wallis; p = 2.1 · 10^–45^). Asterisk indicates significant difference between groups.

We looked at all possible pairings for neurons in our dataset and calculated both signal response similarity (i.e., similarity between the responses to the signal) and noise response similarity (i.e., similarity between the responses to the noise). Figure 4 (main panel) shows the relationship between signal and noise response similarity for On-On pairs (green triangles), Off-Off pairs (magenta triangles), and On-Off pairs (blue circles). We found that signal response similarity values were positive on average for all types of pairs (Fig. 4, y-axis; On-Off: 0.047 ± 0.05; On-On: 0.051 ± 0.054; Off-Off: 0.043 ± 0.043) and significantly different than zero (On-Off: p = 6.83 · 10^–60^; On-On: p = 8.44 · 10^−31^; Off-Off: p = 2.40 · 10^−30^, t-test). Furthermore, the signal response similarity distributions strongly overlapped and did not differ significantly from one another for all pair types (Fig. 4, right panels, Kruskal-Wallis, p = 0.219). It is important to note that signal response similarity values were small on average but significantly different from zero (at p = 0.05; see Methods) for the vast majority of pairs on our dataset (On-Off: 412 out of 420 pairs; On-On: 208 out of 210 pairs; Off-Off: 185 out of 190 pairs;; see Methods).

**Figure 5:**
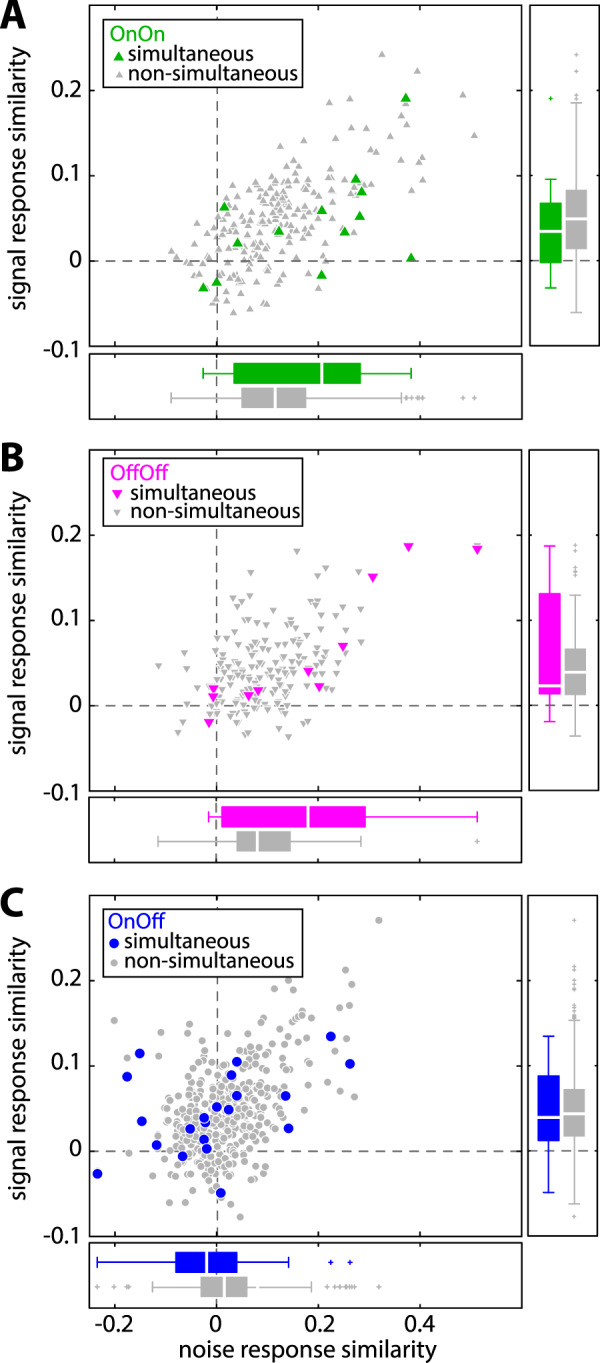
The relationship between signal and noise response similarity did not differ when computed from neuron pairs whose activities were recorded either simultaneously or non-simultaneously. (**A**) Signal response similarity as a function of noise response similarity for same type On-On pairs (simultaneous recordings: green, n = 13; non-simultaneous recordings: gray, n = 210). Distributions of signal response similarity (right, boxplots) and noise response similarity (bottom) were not significantly different from one another (signal response similarity: Kruskal Wallis, p = 0.48; noise response similarity: p = 0.12). (**B**) Same as A. but for same type Off-Off pairs (simultaneous: magenta, n = 11; non-simultaneous: gray, n = 190). Distributions were not different from one another (signal response similarity: Kruskal Wallis, p = 0.82; noise response similarity: p = 0.12). (**C**) Same as A. but for opposite type On-Off pairs (simultaneous: blue, n = 21; non-simultaneous: gray, n = 420). Distributions were not different from one another (signal response similarity: Kruskal Wallis, p = 0.96; noise response similarity: p = 0.17).

We found that noise response similarity values were larger than signal response similarity values in magnitude (On-On: p = 1.0 · 10^−29^; Off-Off: p = 6.6 · 10^−23^) and positive for same-type pairs (Fig. 4, x-axis; On-On: 0.125 ± 0.110; Off-Off: 0.096 ± 0.082), while they were closer to zero for opposite-type pairs (Fig. 2, On-Off: 0.019 ± 0.07). Noise response similarity values were significantly different from zero (at p = 0.05; see Methods) for the vast majority of pairs (On-Off: 385 out of 420 pairs; On-On: 203 out of 210 pairs; Off-Off: 182 out of 190 pairs). As such, population-averaged noise response similarity values were positive and significantly different from zero (On-Off: p = 1.3 · 10^−7^; On-On: p = 1.0 · 10^−40^; Off-Off: p = 1.7 · 10^−37^, t-test). We found that the distribution obtained for opposite-type pairs (blue) was shifted to the left relative to those obtained for same-type pairs (green & magenta). As such, noise response similarity values were on average smaller in magnitude for opposite-type pairs than for same-type pairs (Fig. 4, bottom panels; Kruskal-Wallis; p = 2.1 · 10^−45^). We hypothesized that this decrease in noise response similarity is due to the fact that the responses of On- and Off-type cells to the noise tended to be out of phase and were thus more heterogeneous than those of either On- or Off-type cells. To test this prediction, we computed the similarity between the spike-triggered averages (i.e., the tuning similarity) of individual neurons. Overall, there was a strong positive correlation between tuning similarity and the noise response similarity (Supplementary Fig. S3), confirming our prediction. We further note that the relationships between signal response similarity and noise response similarity was comparable in pairs that were recorded simultaneously and non-simultaneously (Fig. 5).

**Figure 6:**
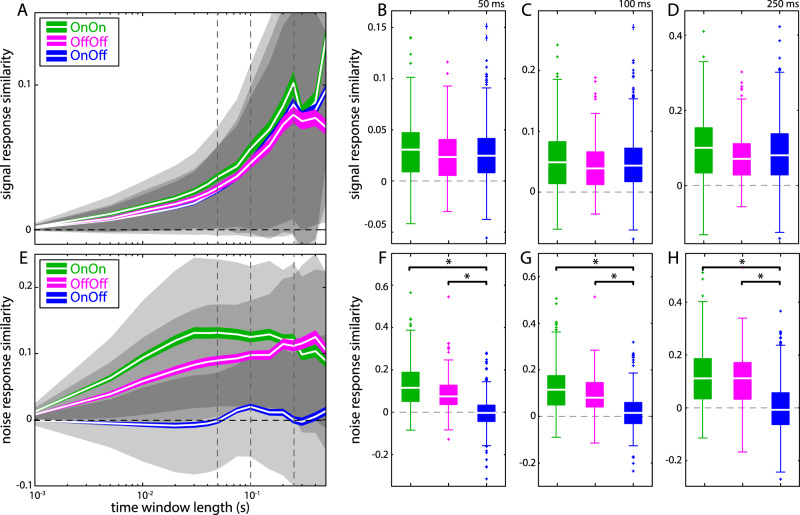
The relationship between signal and noise response similarity was robust to changes in the time window length. (**A**) Signal response similarity values increase as a function of increasing time window length for On-Off (blue), Off-Off (magenta) and On-On pairs (green). White lines depict the mean, colored bands show 1 SEM, gray bands show 1 STD. Vertical dashed lines indicate the time window length for which the signal response similarity values shown in B – D were computed. (**B**) Signal response similarity values obtained for a time window length of 50 ms (mean ± std: On-On, green: 0.030 ± 0.031; Off-Off, magenta: 0.026 ± 0.027; On-Off, blue: 0.026 ± 0.029; Kruskal Wallis, p = 0.137). (**C**) Same as B. but for a time window length of 100 ms (mean ± std: On-On, green: 0.051 ± 0.054; Off-Off, magenta: 0.043 ± 0.043; On-Off, blue: 0.047 ± 0.050; Kruskal Wallis, p = 0.219). (**D**) Same as B. but for a time window length of 250 ms (mean ± std: On-On, green: 0.097 ± 0.098; Off-Off, magenta: 0.079 ± 0.070; On-Off, blue: 0.087 ± 0.088; Kruskal Wallis, p = 0.0776). (**E**) Noise response similarity values increase as a function of increasing time window length for Off-Off (magenta) and On-On pairs (green) but were largely constant and negligible for On-Off pairs (blue). Vertical dashed lines indicate the time window lengths at which the noise response similarity values shown in F – H were taken. (**F**) Noise response similarity values obtained for a time window length of 50 ms (mean ± std: On-On, green: 0.003 ± 0.110; Off-Off, magenta: 0.088 ± 0.085), opposite-type pairs displayed significantly lower noise response similarity values (On-Off, blue: −0.001 ± 0.070; Kruskal Wallis, p = 1.9 · 10^−64^). (**G**) Same as F. but for a time window length of 100 ms (mean ± std: On-On, green: 0.125 ± 0.110; Off-Off, magenta: 0.096 ± 0.082; On-Off, blue: 0.019 ± 0.070 Kruskal Wallis; p = 2.1 · 10^−45^). (**H**) Same as F. but for a time window length of 250 ms (mean ± std: On-On, green: 0.120 ± 0.118; Off-Off, magenta: 0.110 ± 0.104; On-Off, blue: −1.5 · 10^−4^ ± 0.098; Kruskal Wallis, p = 1.1 · 10^−44^). Asterisk indicates significant differences between groups at the p = 0.05 level.

The results obtained above were robust to changes in the time window length used to compute signal and noise response similarity within the interval 1-500 ms (Fig. 6A & E). Specifically, while signal response similarity values were positive on average and did not significantly differ from one another between all types of pairs (Fig. 6B–D), noise response similarity values obtained for opposite-type (On-Off) pairs were closer to zero on average and significantly lower than those obtained for same-type pairs (On-On & Off-Off) (Fig. 6F–H). It is important to note that signal response similarity values increased with increasing time window length (Fig. 6A). This is expected because there is greater variation in the signal over longer time periods.

Thus, our results have shown that all pair types displayed comparable signal response similarity values. This is expected because the response profiles to the signal of single On- and Off-type cells were comparable overall (Fig. 2C). In other words, there was comparable heterogeneity across responses to the signal for the different pair types. In contrast, noise response similarity values were near zero for opposite-type pairs, whereas they were positive for same-type pairs. Again, this is expected because the response profiles to the noise of single On- and Off-type cells were quite different from one another (Fig. 2B). In other words, there was more heterogeneity across responses to the noise when considering both On- and Off-type cells then when considering either only On- or Off-type cells. Specifically, although both On- and Off-type cells encode the slow signal similarly, they encode the faster noise differentially, which leads to lower noise response similarity.

### Heterogeneities increase signal information transmission by neural populations

**Figure 7:**
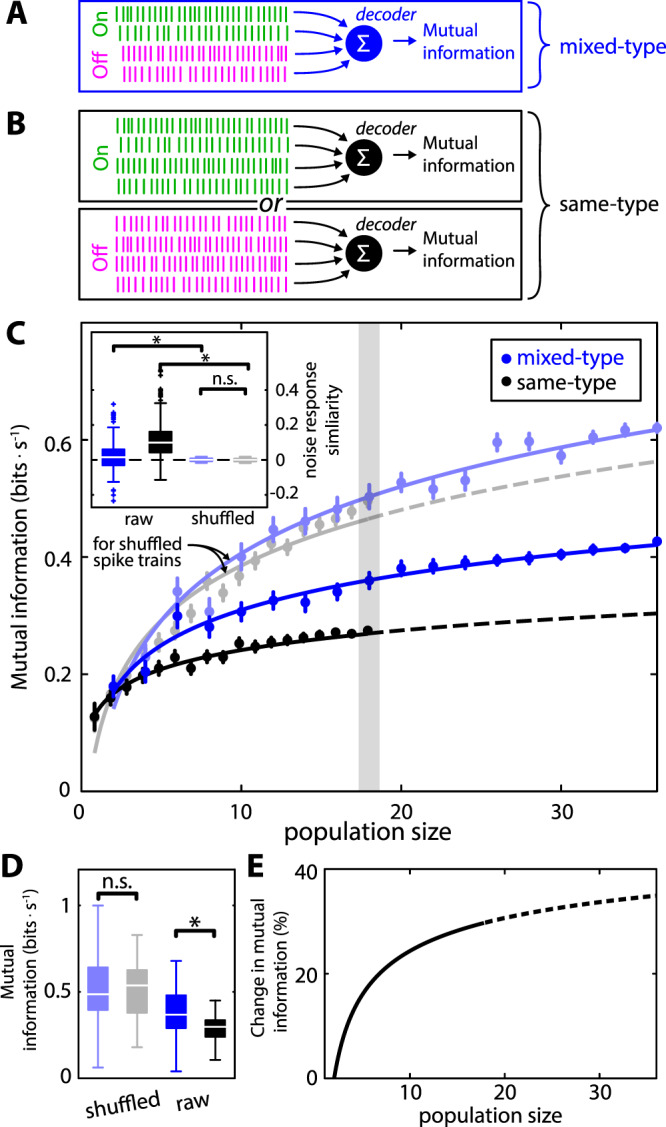
Population coding by ELL pyramidal neuron populations. (**A,B)**. We considered decoders integrating spiking activity from ELL pyramidal neuron populations that were either (**A**) mixed-type (i.e., 50% On- and 50% Off-type), or (**B**) same-type (top: On-type only; bottom: Off-type only) (**C**) Mutual information rate (MI) as a function of population size for mixed-type (dark blue) and same-type (black) populations. The error bars show 1 SEM. Lines are best-fit log functions to the data and were used to extrapolate to larger population sizes for same-type populations (dashed). Light colors indicate MI for mixed-type (light blue) and same-type (gray) populations when shuffling the spike trains with respect to the signal (see below) prior to calculation of MI, which renders the responses to the noise independent of one another. Inset: Noise response similarity for mixed-type (dark blue) and same-type (black) pairs before shuffling. Noise response similarity for mixed-type (light blue) and same-type (gray) pairs after shuffling. Noise response similarity before shuffling was significantly higher for same-type pairs than for mixed-type pairs (p = 1.7 · 10^−5^; Kruskal-Wallis with Bonferroni correction for multiple comparisons; see also Fig. 4). Moreover, while shuffling significantly reduced noise response similarity for both opposite-type and same-type pairs (opposite-type: p = 2.8 · 10^−50^; same-type: p = 1.1 · 10^−86^, Kruskal-Wallis with Bonferroni correction), there was no significant difference between noise response similarity after shuffling obtained for opposite-type and same-type pairs (p = 1.0, Kruskal-Wallis with Bonferroni correction). (**D**) MI values for populations of 18 neurons that are mixed-type (blue: 0.37 ± 0.14 bits · s^−1^) or same-type (black: 0.29 ± 0.06 bits · s^−1^) before shuffling. Also shown are MI values for populations of 18 neurons that are mixed-type (light blue: 0.50 ± 0.20 bits · s^−1^) or same-type (gray: 0.51 ± 0.14 bits · s^−1^) after shuffling corresponding to the gray shading in the main plot of panel C. Before shuffling, MI values were significantly higher for the mixed-type population than for the same-type population (p = 1.1 · 10^−6^, Kruskal-Wallis with Bonferroni correction). After shuffling, MI values were not significantly higher between the mixed-type and the same-type population (p = 1; Kruskal-Wallis with Bonferroni correction). (**E**) Relative difference in MI between mixed-type and same-type population before shuffling as a function of population size (solid) computed from the fits to the data shown in C. The dashed curve is extrapolated from the fitted data.

What are the consequences of the results obtained above on information transmission by neural populations? To answer this question, we considered physiologically realistic decoders that take into account the fact that both On- and Off-type ELL pyramidal neurons make direct excitatory connections with neurons within the midbrain torus semicircularis (TS; Fig. 1B)^36^. Indeed, previous studies have shown that the relative contribution between On- and Off-type ELL input varies considerably across the TS neural population^23^. Specifically, while some TS neurons likely receive primarily input from either On- or Off-type ELL neurons, others likely receive equal amounts of input from both sources. As such, we considered populations that were either mixed-type (i.e., 50% On-type and 50% Off-type; Fig. 7A) or same-type (i.e., all On-type or all Off-type; Fig. 7B). We expect that, for mixed-type populations, the decreased noise response similarity observed for opposite-type pairs as compared to same-type pairs leads to greater independence between the noise responses overall, which then allows for better averaging away the noise responses and therefore increase signal information transmission^37^. The fact that, with increasing population size, the number of possible opposite-type pairs increases at a faster rate than the number of possible same-type pairs (see Material & Methods) also implies that the relative difference between the information transmitted by mixed-type and same-type populations should increase as a function of increasing population size.

**Figure 8:**
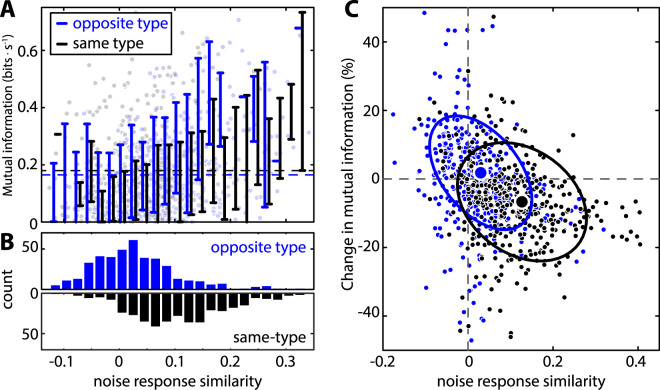
Effects of noise response similarity on information transmission. (**A**) Mutual information for opposite-type (i.e., On-Off, blue) and same-type (i.e., On-On and Off-Off, black) pairs as a function of noise response similarity. The dots show the raw data while the vertical lines show the errorbars (±1 STD) obtained when binning the data using a binwidth of 0.02. In no case there was significant difference between the MI for opposite-type (blue) and same-type (black) pairs (One-way ANOVA, p ≥ 0.14 in all cases). (**B**) Distribution of data shown in A for opposite-type (top, blue) and same-type (bottom, black) pairs. (**C**) Normalized change in mutual information for opposite-type (blue) and same-type (black) neuron pairs before and after shuffling with respect to the signal cycles. In all cases, a significant negative correlation was observed (same-type: r = −0.26, p = 4.3 · 10^−7^; opposite-type: r =−0.33, p = 1.1 · 10^−11^; all data: r = −0.37, p = 1.3 · 10^−25^). The large dots show the mean values and the ellipses show the 64% probability curve for the data (i.e., 64% of the data lies within the ellipse).

Our results confirmed these predictions. Indeed, the mutual information (MI) for mixed-type populations was significantly greater than that obtained for same-type populations with the same size (Fig. 7C, compare dark blue and black curve). As an example, for a population size of n = 18 neurons (Fig. 7D), the resulting mutual information was more than 30% higher for a mixed-type population (i.e., 9 On-type and 9 Off-type cells, dark blue) than for same-type populations (i.e., 18 On-type or 18 Off-type cells, black). Further, the relative difference in information between mixed-type and same-type populations indeed increased with increasing population size (Fig. 7E). It is important to note that this result is primarily if not solely attributable to differences in noise response similarity between same-type and opposite-type pairs. This is because making the noise responses of neurons independent from one another (i.e., setting noise response similarity to zero for all pairs) by shuffling the spike count series with respect to the signal cycle (see Methods; Fig. 7C inset, solid vs. light colors), the mutual information was not significantly different between balanced and unbalanced populations (Fig. 7C main panel & Fig. 7D, light blue vs. gray). Moreover, information from opposite-type and same-type pairs were not significantly different from one another when noise response similarity values were comparable (Fig. 8A,B). Finally, making the noise responses of neurons independent from one another (i.e., setting noise response similarity to zero) through shuffling (see Methods) increased information in neuron pairs when noise response similarity values were positive and decreased information when noise response similarity values were negative (Fig. 8C). Thus, our results show that the reduced magnitude of noise response similarity in On-Off pairs has a significant effect on information transmission when combining them, as occurs in the Torus semicircularis.

## Discussion

We have provided experimental evidence showing that heterogeneities in the responses of a sensory neuron population affect population coding of a slowly time varying signal in the presence of stimulus-induced noise. Specifically, we considered how On- and Off-type ELL pyramidal neurons within the electrosensory system of weakly electric fish responded to a noisy stimulus and its time-varying envelope signal. While single On- and Off-type cells displayed equal levels of heterogeneity in their responses to the signal, they displayed much larger heterogeneities in their responses to the noise. Specifically, single On- and Off-type cell responses were largely in phase with respect to the signal but out of phase with respect to the noise. These differences have important consequences when considering how On- and Off-type cells encode the signal as a population. Indeed, we showed that, while signal response similarity was similar for all pair types (i.e., On-On, Off-Off, and On-Off), there were important differences in noise response similarity. Specifically, On-Off pairs displayed on average less noise response similarity (i.e., greater heterogeneities in their responses) as compared with On-On and Off-Off pairs. As such, when pooling the activities of On- and Off-type cells (i.e., mixed-type population), the lesser noise response similarity displayed by On-Off pairs relative to same-type (i.e., On-On and Off-Off) pairs implies that their noise responses are more independent of one another, which allows for better averaging of these responses and thereby increase signal information transmission.

### Impact of neural heterogeneities on population coding of behaviorally relevant electrosensory stimuli

We note that the effect of increased heterogeneity in the responses of On- and Off-type neurons to the stimulus-induced noise is best seen when pooling the activities of On- and Off-type neurons in a mixed-type population as compared to a same-type population of the same size (Fig. 7C). This is because mixed-type ELL populations display more heterogeneities (i.e., less similarity or more independence) in their responses to the stimulus-induced noise, which is beneficial as it allows for better averaging out the noise when pooling neural activities. Moreover, since On- and Off-type cells displayed on average similar responses to the signal, the benefit of increased heterogeneities in responses to the stimulus-induced noise is not mitigated by increased heterogeneities in responses to the signal, thereby allowing for increased information transmission. We note that such a decoding scheme is physiologically realistic in the electrosensory system given that previous studies have shown that TS neurons receive excitatory input from both On- and Off-type ELL pyramidal neurons^23,36^. Specifically, some “On-Off” TS neurons receive inputs that are matched in strength from On- and Off-type neurons, while other TS neurons receive input predominantly from either On- or Off-type neurons^23,24,38,39^. We hypothesize that these “On-Off” neurons will best respond to the signals considered in the present study and thus mediate the animal’s behavioral responses. Further studies are needed to verify this prediction.

We also note that the decoding scheme considered here is linear since we summed the neural activities. Previous studies have shown that TS neurons integrate input from ELL in a nonlinear fashion, which likely contributes to increased response selectivity^40,41^. As such, the information estimates reported here constitute a lower bound on the information available to downstream areas^42,43^. Future studies should thus investigate how encoding by TS neurons are influenced by nonlinear integration of afferent ELL input from On- and Off-type pyramidal neurons. In particular, the location of receptors within the dendritic tree as well as the presence of subthreshold membrane conductances such as T-type calcium channels^41^ should be investigated. This is because these nonlinearities could further decrease the similarity (or, equivalently, make them even more independent) between the inputs from ELL pyramidal neurons in response to the stimulus-induced noise, as seen in other systems^44–46^. It is important to note here that it is not expected that electrosensory neural circuits will explicitly compute similarities between neural responses to the signal or the stimulus-induced noise. Rather, our results suggest that, by combining inputs that are matched in strength from On- and Off-type ELL pyramidal neurons, electrosensory circuits can significantly reduce the deleterious effects of similarities in responses to the stimulus-induced noise, which is detrimental to averaging when pooling neural activities. It is important to note that On- and Off-type ELL pyramidal neurons also respond to other stimuli, such as those caused by objects whose conductivity is greater or lesser than that of the surrounding water. Specifically, On- and Off-type cells are excited by objects whose conductivity is greater and lesser than that of the surrounding water, respectively^47,48^.

What causes differences in the level of heterogeneities within the responses of On- and Off-type cells to the signal and the stimulus-induced noise considered here? Response heterogeneities within the pyramidal neuron population arise in part due to strong descending input from higher brain regions. Previous studies have shown that descending input can strongly affect single ELL pyramidal neuron responses not only to the signal but also to the stimulus-induced noise^29,30,49,50^. Further studies are needed to determine how descending input mediate coding by the ELL pyramidal neuron population.

Finally, it is important to point out that the current study did not take into account correlations between neural activities that arise from shared synaptic input^46^. Indeed, these so-called “noise correlations” can impact population coding by introducing either synergy or redundancy^51–61^. Noise correlations can only be assessed by recording neural activities simultaneously^62^. Previous studies have shown that ELL pyramidal neurons display noise correlations that most likely originate from shared afferent input^63,64^. However, these noise correlations are “local” in that their magnitude decreases with decreased receptive field overlap. The fact that similar levels of signal and noise response similarity were observed between simultaneously and non-simultaneously recorded pairs of neurons in our dataset suggests that noise correlations might not strongly impact population coding in the context considered here. We hypothesize that this is because the signal and stimulus-induced noise considered here are “global” because they recruit most if not all of the pyramidal neuron population. Previous studies have shown that noise correlation magnitude is reduced when “global” stimuli are presented^63,65^, which supports our hypothesis. It is nevertheless important to point out that noise correlations are likely to have a much stronger impact on the coding of other behaviorally relevant stimuli, such as those caused by prey. Further studies are however needed to verify this prediction.

### Implications for other systems

Our results are likely applicable to population coding in other sensory systems. This is because behaviorally relevant slowly varying envelope signals such as the one considered here are also found across various modalities (visual^21,66^, vestibular^67^, auditory^68^; mechanosensory^69^). Importantly, these slowly varying envelope signals are independent of other stimulus features, which can thus be considered noise (auditory^70,71^; visual^72^; vestibular^67,73^). On- and Off-type neurons are also found across multiple sensory modalities (visual^74–77^:, chemosensation^78^, thermosensation^79^, audition^80^, vestibular^81^) and multiple functional roles have been proposed for having both cell types such as metabolically efficient signaling of opposite changes in the stimulus^82,83^ and improving the representation of natural signals^74^. We predict that On- and Off-type neurons in other systems will also demonstrate better encoding of a slow varying signal in the presence of stimulus-induced noise because their response heterogeneities to the noise are greater than those to the signal, as demonstrated here for the electrosensory system.

Future studies should therefore take into account noise response similarity and examine their effects on population coding. This can be achieved by using stimulation paradigms in which the envelope signal and the stimulus-induced noise are independent of one another (i.e., the signal is repeated across trials but not the stimulus-induced noise), as in the current study. This stimulation paradigm differs from those typically used for which instead both the envelope signal and the stimulus-induced noise would be repeated from trial to trial. We argue that the former stimulation paradigm is more realistic than the latter because it better mimics the known structure of natural stimuli and the fact that higher order neurons can respond to the envelope signal independently of the stimulus-induced noise. Such neurons are found not only in the electrosensory system^23^ but also in the visual^84^ as well as auditory^85^ systems. As such, we predict that population coding will most likely be influenced by the stimulus-induced noise as studied here.

## Material & Methods

### Animals

Specimens of *Apteronotus leptorhynchus* were obtained from tropical fish suppliers and housed in groups of up to 15 (water temperature 29 ± 2 °C; water conductivity of 100–300 µS · cm^−1^) as per published guidelines^86^. All housing and experimental procedures were approved by McGill University’s animal care committee according to the guidelines of the Canadian Council on Animal Care. All procedures were carried out under animal use protocol number 5285.

### Experimental Design and recording

Electrophysiological recordings were made from the hindbrain electrosensory lateral line lobe (ELL) of N = 12 fish (13 ± 2 cm). Animals were immobilized with an intramuscular injection of tubocurarine chloride hydrate (Sigma-Aldrich; 200 µl injection; 2 mg · ml^−1^) and respirated with a constant flow of water over their gills (≈ 10 ml · min^−1^). After topical application of local anaesthesia (2% lidocaine, Western Medical Supply, Arcadia, CA, USA), a small craniotomy (≈ 5 mm^2^) was made in the dorsal skull to access the hindbrain. Recordings were performed with two metal-filled recording micropipettes (0.5–1 MΩ) inserted into the lateral segment (LS) of the ELL. Electrode signals were amplified and filtered (x1000, 300–5 kHz; AM differential amplifier 1700; AM Systems, Sequim, WA USA), digitized at 20 kHz (CED Power 1401, Cambridge Electronic Design, Cambridge, UK), and stored for further analysis (Spike II, v7.16 ×86, CED). Recordings were either performed simultaneously (i.e. one isolated unit on each of the two recording electrodes simultaneously; n = 68 neuron pairs) or non-simultaneously (i.e. one isolated unit on only one of the recording electrodes; n = 50). We chose to record from pyramidal neurons within LS because these display the largest receptive fields^87,88^ and the greatest sensitivity to both the stimulus waveform^89^ and its envelope signal^26^. As such, we expect that neural responses to the stimulus waveform will have the greatest impact when considering population coding of the envelope signal in this segment.

Under immobilization, *Apteronotus leptorhynchus* specimens continue to emit the EOD signal due to the neurogenic nature of their electric organ. As such, they remain capable of displaying electrosensory behaviors consisting of changes in the EOD frequency (Fig. 1C,D). Stimuli consisted of amplitude modulations of the animal’s self-generated electric organ discharge, applied by multiplying (MT3 analog multiplier, Tucker-Davis Technologies, Alachua, FL USA) the desired waveform (described below) with a train of single-cycle sinusoidal waveforms that were phase-locked to the animal’s EOD^90^. For this, the EOD zero-crossings were detected (121 Window discriminator, World Precision Instruments WPI, Sarasota, FL USA) to trigger a waveform generator (33220 A LXI arbitrary waveform generator, Agilent, Santa Clara, CA USA) that generated one cycle of a sinewave per EOD cycle. The output of the multiplier was isolated from ground (A395 linear stimulus isolation unit, WPI) and delivered to the animal via two steel wire electrodes located about 15 cm from each side of the animal. Our stimuli consisted of zero-mean low-pass filtered (15 Hz cut-off frequency, 8^th^ order Butterworth) Gaussian white noise (i.e., the noise) with a duration of 90 s, whose amplitude (i.e., envelope) was modulated sinusoidally at 1 Hz (the signal). The noise was independent of the envelope by construction (see Supplementary Fig. S2). While the sinusoidal envelope stimulus used in this study is not naturalistic, we note that it has been previously shown that responses of ELL pyramidal neurons to sinusoidal envelopes can be used to predict their responses to natural envelope waveforms^27^. Stimulus contrast was adjusted to 15–20%. For data analysis (described below), each envelope cycle was considered one repetition (Fig. 1A) and the low-pass filtered white noise was not repeated from cycle to cycle. The same stimulus waveform was presented to all neurons.

### Data Analysis

For behavior, the recorded times at which the EOD crosses zero from below were converted into a series of events with 30 kHz sampling rate. For visualization purposes, this series of events was low pass filtered (2^nd^ order Butterworth filter, 3 Hz cutoff) and detrended. We used linear systems identification techniques to assess behavioral gain and phase as done previously^15,25,27,28,91^. Specifically, gain was calculated as:

1 $$gain=\frac{{A}_{behavior}}{{A}_{envelope}}$$

where *A*_*behavior*_ is the amplitude of the EOD frequency modulation as determined by fitting a sinusoid to the average time-varying EOD frequency during one envelope cycle and *A*_*envelope*_ is the amplitude of the electrosensory envelope as extracted from a local dipole electrode (1 mm tip spacing) that was placed close to the skin of the animal. We further determined the phase shift between the behavioral responses and the envelope stimulus as:

2 $$\theta =2\pi \frac{{T}_{\max (E)}-{T}_{\max (EOD)}}{{T}_{{\rm{\max }}(E)}}$$

where T_max_ denotes the times at which either the envelope (E) or the EOD frequency (EOD) reach their maximum values during the envelope cycle.

For neuronal data, spike times were detected from the recorded traces using a threshold and assigned to different neurons based on waveform, interspike intervals, and PCA with subsequent k-means or normal-mixtures clustering (Spike II, CED). Further analyses were carried out using Matlab (MATLAB R2015b v8.6.0, MathWorks Inc., Natick, MA, US). Specifically, for each neuron, spike times were converted into a binary sequence with a bin width of 0.5 ms. The binwidth was chosen to be smaller than the absolute refractory period of ELL pyramidal neurons (~ 2 ms) such that at most one spike can occur within any given bin. Time-dependent firing rates were obtained by low-pass filtering the binary sequence (3 Hz cut-off frequency, 2^nd^ order Butterworth) and multiplying by the sampling frequency of 2000 Hz. Negative values of the firing rate were set to zero.

In order to test that a neuron responded significantly to the envelope signal, we computed the vector strength^92^ as done previously^26^. Only neurons with a vector strength higher than 0.085 were used for further analysis (single neurons: n = 41; neuron pairs: n = 46). Vector strength values for neurons included in the analysis ranged between 0.0859 and 0.4503.

We categorized recorded cells into either On- or Off-type by using their responses to an AM stimulus waveform as done previously^28^. Importantly, for this we used a stimulus (Gaussian white noise, 120 Hz cutoff frequency, 8^th^ order Butterworth low-pass filter) that is independent from the one used to investigate population coding. Specifically, we computed the spike triggered average (STA) stimulus waveform and determined the time-averaged STA slope within an evaluation window (8 ms wide) centered 8 ms before the trigger point (t_0_) to account for the average action potential transmission delay from the skin surface to the hindbrain ELL. Neurons for which the STA slope was on average positive during the evaluation window were classified as On-type whereas those for which the STA slope was negative were classified as Off-type (Fig. 2). We furthermore quantified tuning similarity by computing the correlation coefficient between the STA waveforms for each neuron pair.

The average phase difference between the binary sequence and the 0–15 Hz noise was computed as a function of frequency *f* using^23^:

3 $$\phi (f)=arctan\left(\frac{imag[{P}_{rs}(f)]}{real[{P}_{rs}(f)]}\right)$$

where *arctan* denotes the arctangent, while *imag* and *real* denote the imaginary and real parts, respectively. *P*_*rs*_*(f)* is the cross-spectrum between the noise and the binary sequence computed using the function “cpsd” in Matlab. The phase difference was then averaged over the frequency range 0–15 Hz.

The average phase difference between the neural response and the signal was calculated by fitting a sinusoidal function to the phase histogram obtained over 90 consecutive cycles of the envelope cycle (i.e., each spike time was converted to a phase based on where it occurred during the cycle of the sinusoid as done above for the EOD zero crossings). Response phase was determined as the phase for which the fitted function was maximum as done previously^26^.

We quantified the similarity between responses by the correlation coefficient between pairs of spike count sequences. Spike count sequences were obtained from each spike train by counting the number of spikes occurring during successive and non-overlapping time windows that were always aligned with respect to the sinusoidal signal and whose length ranged between 1 and 250 ms. Spike count sequences were tested for stationarity using an augmented Dickey-Fuller test (function “Adftest” in MATLAB). For all data, the null hypothesis of nonstationarity could be rejected (p < 10^–3^ in all cases). We then computed the correlation coefficient between the two spike count sequences using Pearson’s correlation coefficient:

4 $$r=\frac{Cov({n}_{1}{n}_{2})}{\sqrt{Var({n}_{1})Var({n}_{2})}}$$

To compute signal response similarity, we considered the segments of the spike count sequence obtained during each signal cycle and randomly permuted their order to obtain shuffled spike counts. It is important to note that this shuffling procedure does not affect the envelope signal itself. The signal response similarity was quantified as the correlation coefficient between the shuffled spike counts for each neuron pair as per Eq. (4) averaged over 20 independent realizations of the shuffling procedure. It is important to note that signal correlations will trivially be null when the duration of the time window is equal to the signal period (1 s), this is because the signal does not vary over that time window length by definition. We thus considered time windows whose length was up to a quarter of the envelope cycle. We did not use the filtered firing rates (Fig. 4) during analysis because low-pass filtering with a cutoff frequency of 3 Hz greatly attenuates the stimulus-induced noise whose impact on information transmission we are studying. We note that using a time window length of 100 ms would roughly correspond to a cutoff frequency of 10 Hz. For each pair, significance was assessed by computing signal response similarity for surrogate datasets in which the binary sequences for that pair were randomized in order to eliminate any correlation between then (i.e., signal response similarity should be zero in theory). An experimentally obtained value of signal response similarity without shuffling was deemed to be significant if that value was greater in magnitude than the 95% confidence interval of the signal response similarity value distribution obtained from 100 surrogate datasets.

Finally, noise response similarity was quantified as the correlation coefficient between the spike count residual sequences. The spike count sequences were first averaged over signal cycles (Fig. 3A) and the mean spike count sequence was then subtracted from the spike counts for each cycle to obtain the residual spike counts sequences (Fig. 3B). Significance for noise response similarity was assessed in a manner analogous to that described above for signal response similarity.

To investigate the effects of neural correlations on information transmission, we computed the mutual information rate (MI) between the signal and the summed binary sequences for a given neural population using^42^:

5 $$MI=-\int dflo{g}_{2}\left[1-\frac{{|{P}_{rs}(f)|}^{2}}{{P}_{rr}(f){P}_{ss}(f)}\right]$$

where *P*_*rs*_*(f)* is the cross-spectrum between the stimulus and the summed response computed using the function “cpsd” in Matlab, *P*_*rr*_*(f)* is the summed response power spectrum computed using the function “pwelch” in Matlab, and *P*_*ss*_*(f)* is the signal power spectrum computed using the function “pwelch” in Matlab. We considered “same-type” populations consisting either of *n* On-type or Off-type neurons only, or “mixed-type” populations for which *n/*2 neurons were On-type and the remaining *n/*2 were Off-type. We note that for a mixed-type population of *n* neurons, the number of same-type pairs (On-On + Off-Off) is given by *n*^*2*^*/4-n/2*, while the number of opposite-type pairs (On-Off) is given by *n*^*2*^*/4* respectively. As such, with increasing *n*, the number of opposite-type pairs in the population will always be greater than the number of same-type pairs and their difference, which is equal to *n/2*, grows linearly with population size *n*. For a given population size, results were obtained from random re-combinations of neurons from our dataset and were averaged over the highest number of possible iterations up to 200. Plots of mutual information as a function of population size were fitted using either *A* log(*n*) or $A\sqrt{n}$. Overall, the logarithm function gave better fits to the data as determined from rmse values ranging between 0.0099–0.0272 (log) vs. 0.0148–0.109 (sqrt). As such, we only show the fit for the log function in the figure. Mutual information rates were also computed after randomly shuffling the segments of binary sequences with respect to the signal cycles, such as to make the responses to the noise independent of one another across the population.

### Statistical Analysis

Statistical significance was assessed using Kruskal-Wallis non-parametric tests, one sample or paired sample or Wilcoxon signrank tests or paired t-tests. P-values are given in the text and figure legends. In all figures, asterisk indicates statistical significance. Values are reported as mean ± std throughout the text unless otherwise stated.

## Data Availability

The datasets generated during and/or analysed during the current study are available in the figshare repository at https://doi.org/10.6084/m9.figshare.10380131.

## Supplementary.information

Supplementary.information.
