# Supplementary material for: Neuronal On- and Off-type heterogeneities improve population coding of envelope signals in the presence of stimulus-induced noise
Source: Sci Rep. 2020 Jun 23;10:10194. doi: 10.1038/s41598-020-67258-1 (PMC7311526; doi:10.1038/s41598-020-67258-1)
Supplement: Supplementary file 1 — Supplementary.information. [file 41598_2020_67258_MOESM1_ESM.docx]

**Supplementary Material** for manuscript entitled
*“**Neuronal On- and Off-type heterogeneities improve population coding of envelope signals in the presence of stimulus-induced noise”.*

**Authors:** Volker Hofmann, Maurice J Chacron

**
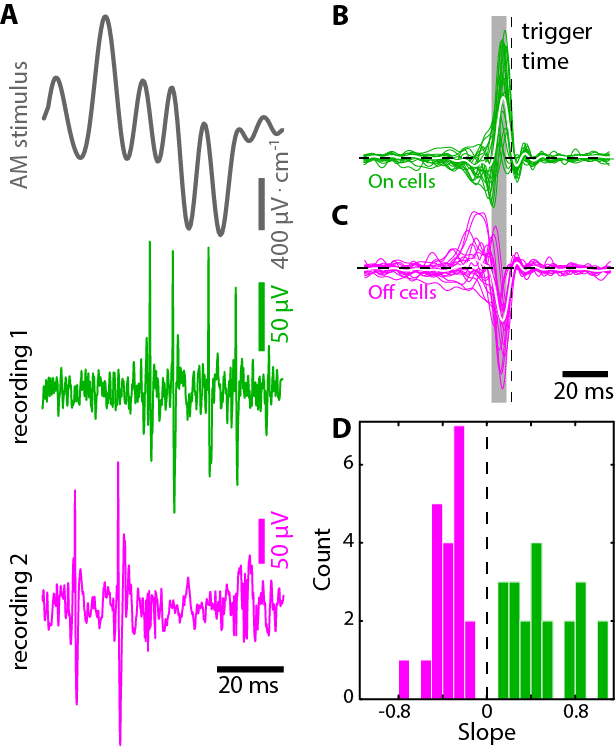
**

**Supplementary Figure S1: Classification of cells as either On- or Off-type. A.** A segment of the broadband (0 – 120 Hz) AM probe stimulus (gray, top) and extracellular recordings showing the spiking responses of example On-type (green, middle) and Off-type (magenta, bottom) ELL pyramidal neurons. **B.** Spike-triggered averages (STAs) of On-type cells. The white line shows mean of all STAs. Gray shading depicts the time window during which the STA slope was evaluated. The vertical dashed line at t_0_ denotes the action potential time. **C.** Same as B, but for Off-type cells. **D.**Histogram of average value of the STA slope during the time window depicted by the grey area in B & C. Cells for which the STA slope was positive were classified as On-type (green, n = 21) whereas those for which the STA slope was negative were classified as Off-type (magenta, n = 20).

**
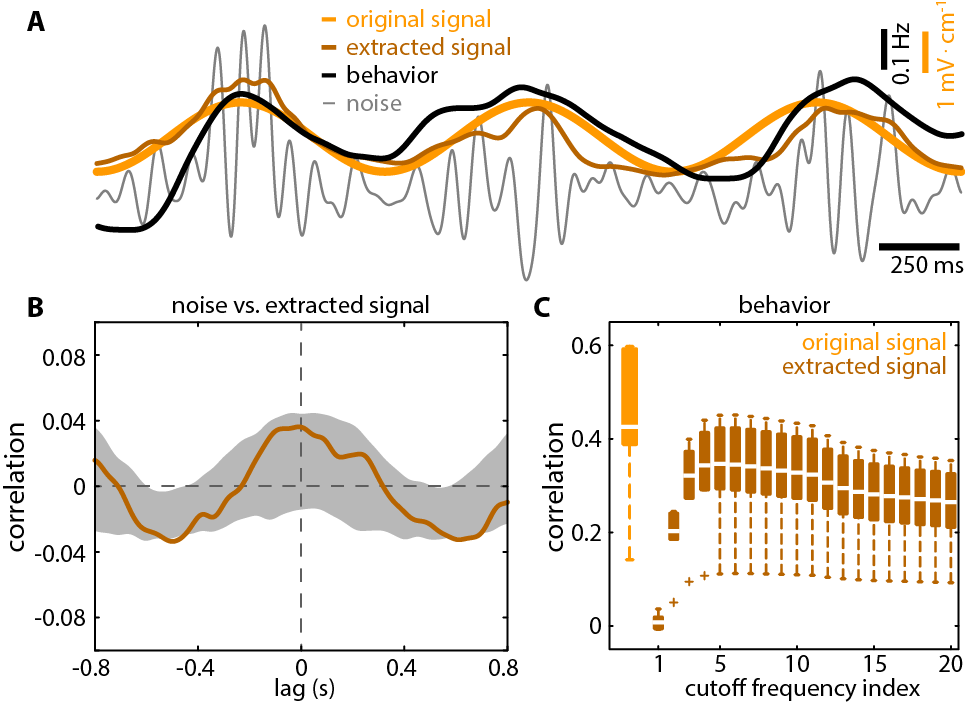
**

**Supplementary Figure S2: The stimulus and its envelope signal are independent of one another. A.** Time series showing the stimulus noise (gray) together with the original sinusoidal signal (orange) as well as that extracted from the stimulus waveform (brown). The animal’s behavioral response (black) is also shown and was aligned to the signals for illustration purposes. The extracted signal was obtained using a standard nonlinear transformation (Hilbert transform) followed by low-pass filtering (1^st^ order Butterworth with 2.5 Hz cutoff frequency). **B.** Cross-correlogram between the stimulus noise and the extracted signal. The gray band shows the error around the estimated values (95% confidence interval obtained from 100 iterations of 20% jackknifing of the data). **C.** Population-averaged correlation coefficients between behavioral responses and the signal (orange, left) and the extracted signal (brown, right) for different cutoff frequencies (cutoff frequency index 1 – 20 correspond to cutoff frequencies of 0.1, 0.5, 1, 1.5, 2, 2.5, 3, 3.5, 4, 4.5, 5, 7.5, 10, 12.5, 15, 17.5, 20, 25, 30, 50 Hz). Correlation coefficients between behavioral responses and the signal were significantly higher than those between behavioral responses and the extracted signal for all cutoff frequencies (paired t-test, p = 6.2 · 10^-3^; N = 7). For this analysis and presentation in panel A, behavioral responses were shifted such as to be in phase with either the sinusoidal or the extracted signal using standard linear systems analysis techniques.


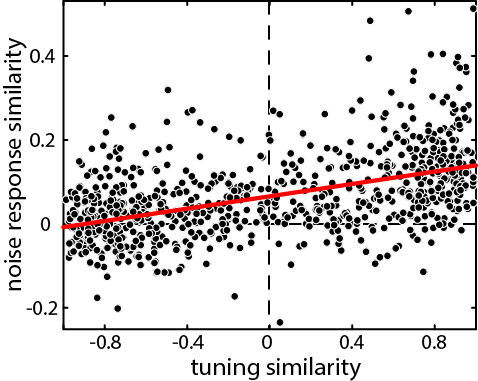


**Supplementary Figure S3: Noise response similarity is positively correlated with tuning similarity.**

Noise response similarity (computed for a time window length of 100 ms) as a function of tuning similarity (i.e., similarity between the STA waveforms of neuron pairs; See Methods). A significant positive correlation was observed (R = 0.50, p = 2.5 · 10^-52^; N = 820).
